# Reducing Peripherally Inserted Central Catheter Tip Migration in Neonates: A Proactive Approach to Detection and Repositioning

**DOI:** 10.3390/jcm14061875

**Published:** 2025-03-11

**Authors:** Debbie Diewo, John Mawson, Sandesh Shivananda

**Affiliations:** 1 Department of Pediatrics, Sarawak General Hospital, Kuching 93586, Malaysia; debbiediewo@moh.gov.my; 2 Department of Pediatrics, University of British Columbia and BC Women’s and Children’s Hospital, Vancouver, BC V6H 3N1, Canada; 3 Department of Radiology, University of British Columbia and BC Women’s and Children’s Hospital, Vancouver, BC V6H 3N1, Canada; jmawson@cw.bc.ca

**Keywords:** peripherally inserted central catheter, migration, reposition, surveillance

## Abstract

**Objective**: Peripherally Inserted Central Catheter (PICC) tip migration often occurs after placement despite efforts to position the tip centrally. This study aimed to quantify PICC tip migration within 3–7 h post-insertion and evaluate the effectiveness of manual adjustments for repositioning. **Methods**: This single-centre retrospective study evaluated the impact of a proactive approach following PICC placement, which included standardized body positioning during X-rays, defined target PICC tip locations, radiological surveillance at 4–6 h post-insertion, and guided manual adjustments. We included all infants receiving PICCs during a five-year period; trained nurses and physicians in vascular access performed the insertions. **Results**: Of 712 infants included, the median gestational age was 30 weeks, and the median birth weight was 1386 g. PICC tip migration occurred in 211 infants (30%) within 3–7 h post-insertion, with 81% of cases involving inward migration into the cardiac silhouette. Migration was more common in upper limb PICCs (82%). Manual adjustments achieved satisfactory repositioning in 191 infants (83%). None of the infants experienced pericardial effusion. **Conclusions**: A proactive approach that standardized imaging protocols, timing, and PICC tip positioning detected migration in 30% of infants and successfully facilitated repositioning in 83% of cases.

## 1. Introduction

Neonates in the NICU often receive peripherally inserted central catheters (PICCs) for parenteral nutrition and medication delivery. Compared to peripheral intravenous catheters (PIVs), PICCs offer many benefits, including reduced pain, minimized handling during peripheral intravenous cannulation, the ability to deliver concentrated fluids or nutrients, bedside placement in the neonatal intensive care unit (NICU), and extended use over weeks or months [1,2]. Despite these advantages, PICCs in neonates are associated with life-threatening complications, including pericardial, pleural, and peritoneal effusion, pericardial tamponade, dysrhythmias, and other issues such as occlusion, breakage, leakage, and infections [2,3,4]. These complications occur in 0.2–10% of cases [2,3,5,6,7]. The life-threatening complications are more likely to occur when PICC tips are in the right atrium [2,3,4,5,8,9,10].

Even with clinicians’ best efforts to position the PICC tip centrally, migration may occur as a result of arm movement, vein selection, changes in catheter course after infusion, blood flow dynamics, and soft tissue compression around the catheter [7,11]. These variables, coupled with infant-specific characteristics, necessitate close monitoring. Surveillance within 24 h of insertion is strongly recommended to detect migration [4,6,11,12].

In 2015, we experienced three PICC-related life-threatening events, including pericardial effusion and tamponade, despite verifying tip placement with immediate post-insertion X-rays. Following a critical review of these events, we implemented a proactive approach that standardized body positioning during X-rays, defined target PICC tip locations, conducted radiological surveillance 4–6 h post-insertion, and provided guidance for manual adjustments. However, it remains unclear whether early interventions during the first few hours of PICC placement increase the likelihood of maintaining central or low-risk, non-central tip positions. The study focuses on outcomes following the adoption of a proactive approach designed to enhance the safety and accuracy of PICC tip positioning in neonates.

### Objectives

This study aims to quantify the frequency of PICC tip migration within the critical 3–7 h post-insertion and assess the effectiveness of manual adjustments in repositioning the tip to achieve central or low-risk, non-central tip positions.

## 2. Materials and Methods

### 2.1. Design and Setting

We conducted a retrospective cohort study on infants admitted to the neonatal intensive care unit (NICU) at BC Women’s Hospital, Vancouver, between January 2017 and December 2021. Nurses, clinical associates (pediatricians), or neonatal fellows with vascular access training inserted the PICCs. During the study period, three types of catheters were available: NutriLine 2 Fr single- and double-lumen polyurethane catheters, Premicath 1 Fr catheters (Vygon, Écouen, France), and L-Cath™ 1.2 Fr polyurethane catheters (Argon Medical Devices, Plano, TX, USA). The choice of catheter and insertion site depended on the operator’s preference.

### 2.2. Proactive Approach (Interventions)

A proactive and systematic method ensures optimal placement and positioning of PICCs. This approach emphasizes proper limb and body positioning, precise determination of target positions for the PICC tip, manual adjustments informed by X-ray imaging, and vigilant early radiological surveillance (Supplementary Figures S1 and S2). Three X-rays in anteroposterior view were performed within the initial six hours, with the first two being done shortly after PICC placement. The first X-ray determined the PICC tip position, the second confirmed its deepest placement, and the third was conducted as part of routine radiological surveillance between 3 and 6 h. The first image captures the limb in positions commonly adopted during the insertion process. For upper limb PICCs, this involves abducting the shoulder and extending the elbow, while for lower limb PICCs, the hip is abducted and the knee extended. For scalp PICCs, the head is turned away from the insertion site [7,13]. Next, we adjust limb positions to promote inward PICC tip movement and then take a second image while the insertion team maintains full aseptic precautions, thereby allowing for both advancement and retraction of the PICC as needed [7,13]. For upper limb PICCs, this entails adducting the shoulder and flexing the elbow. For lower limb PICCs, flexing the hip and knee into a frog-like posture. For scalp PICCS, the head is positioned in the midline, chin to chest. The precise anatomical location of the PICC tip is critical for its efficacy and to minimize complications. For upper limb and scalp PICCs, the target location is within the superior vena cava (SVC), which corresponds to vertebral levels T3 to T7. For lower limb PICCs, the target location of the tip was within the inferior vena cava (IVC), aligning it with vertebral levels T9 to L1 or L3 to L5 while avoiding the L2 region to prevent interference with the renal vein junction [13,14]. Post-insertion X-rays provide essential guidance for adjusting the PICC line’s position. These images allow the clinician to estimate the required length for advancing or retracting the PICC to achieve optimal placement. Clinicians carefully ensure the PICC tip does not extend beyond T7 for upper limb and scalp PICCs or beyond T9 for lower limb PICCs. If, despite positional adjustments, the PICC tip remains in a non-central location, the care team evaluates the situation to decide the best course of action. Depending on the difficulty, urgency, and feasibility of alternative intravenous access, the team may choose to replace the line, temporarily use the PICC in a suboptimal position with or without arm manipulation [7], or retract it into the subclavian, axillary, jugular, iliac, or femoral vein. To reduce the risk of complications, the team avoids keeping the catheter tip near flexion creases (i.e., axilla or inguinal area) and in the brachiocephalic vein with the PICC tip not sloping downwards toward the SVC [14,15]. Radiological surveillance is an integral component of the proactive approach, ensuring proper PICC tip positioning after insertion. We image the patient 4 to 6 h post-insertion (3rd image), positioning their limb, head, or neck in the second view posture previously described to visualize the deepest PICC tip position. This 3rd image helps detect any migration of the PICC tip and allows for timely manual repositioning, either to central locations or to safe, non-central positions when necessary.

We anchored the catheter wings to the skin with small Steri-Strips to prevent inward movement. We coiled the excess catheter tubing near the insertion site to minimize tension and inadvertent movement. To secure the catheter and protect it from contamination and moisture, we applied a transparent dressing (Tegaderm™, 3M, St. Paul, MN, USA) over the insertion site. Finally, we covered the catheter hub with an additional dressing.

We did not use ultrasound guidance for PICC insertions. All PICCs received a continuous infusion of heparin at 1.0 unit/mL at a rate of 1 mL per hour, following the standard unit policy. If repeated attempts to insert a PICC were unsuccessful, interventional radiologists performed the procedure under fluoroscopic guidance. Clinicians documented details of catheter insertion, removal, and status of the catheter site and infusate volume in the patient charts. Nurses monitored the PICC dressing daily and replaced it when it became loose or soiled. The attending neonatologist decided whether to insert or remove a PICC based on clinical needs. Nurses removed catheters upon completion of intravenous therapy or sooner if complications arose. Because of the small size of central catheters used in NICU infants, obtaining blood samples via the PICC was often impractical.

### 2.3. Implementation (Plan-Do-Study-Act Cycle)

After developing the guideline, we provided orientation to PICC team members (n = 10) in a two-hour workshop. The workshop included a didactic lecture, mannequin simulations for limb and body positioning, review of X-rays, and annotation of X-rays to standardize body positioning during X-rays, define target PICC tip locations, conduct radiological surveillance at 3–6 h, and perform manual adjustments. Radiology technicians conducting X-rays and point-of-care staff (respiratory therapists and nurses) holding infants during X-rays received a one-hour orientation on body positioning during the annual spring education days in 2016. All physicians, including neonatologists, radiologists, neonatal fellows, and clinical associates, received orientation on target PICC tip positions and guided PICC team members when requested to review the PICC tip position following the X-rays in their administrative meetings. We incorporated the workshop into the onboarding process for new PICC team members and added body positioning training to the orientation package for new staff.

### 2.4. Inclusion and Exclusion Criteria

We included only PICCs inserted during an infant’s stay in our unit. To avoid errors from repeated measures in a single patient, we considered only the first PICC for each infant. We excluded infants with PICCs placed in other hospitals or units, those with suboptimal limb or head positioning during X-rays, and those with surveillance X-rays taken less than 3 h or more than 7 h post-insertion. To minimize unnecessary handling, we selected a broader 3–7 h time frame to accommodate infants whose X-rays were performed outside the recommended 4–6 h window because of procedural clustering.

### 2.5. Data Collection

We identified infants with PICCs from our unit’s database housed within the Canadian Neonatal Network (CNN) platform [16]. Details such as day of life, postmenstrual age, insertion site, and inserter’s profession were obtained from electronic patient records. We reviewed images and X-ray reports from the Picture Archiving and Communication System (PACS) to determine PICC tip location and migration at four time points: (1) immediately post-insertion, (2) 3–7 h post-insertion, (3) on the first X-ray performed after 7 days post-insertion, and (4) on the final X-ray prior to PICC removal, if multiple X-rays were available. While we followed unit guidelines for X-rays at time points 1 and 2, later X-rays were obtained for non-PICC-related reasons. Follow-up X-rays after manual adjustments, if required, were at the care team’s discretion. The radiologist investigator (JM) ensured a 90% or higher inter-rater agreement with investigator (DD) using 40 infant images. We collected data on infant demographics, discharge outcomes (mortality, morbidity, length of stay), and PICC-related adverse events from the CNN database and the patient safety learning system, a web-based tool to report and learn from patient safety incidents and hazards.

### 2.6. Definitions

We defined “central” catheter tip position as being in the superior vena cava (SVC) or inferior vena cava (IVC) corresponding to vertebral levels T3–T7 and T9–L1 or L3–L5, respectively. Non-central tip position was defined as a PICC located in the subclavian, axillary, brachiocephalic, and iliac veins or within the right atrium beyond the SVC/IVC [13,14]. We defined migration as any change in tip position from the baseline between central and non-central locations, assuming similar limb or neck positions between images [2,6,7,11,12]. We identified four migration patterns. Central to non-central (Inward): The tip extended beyond the SVC-right atrium (RA) junction into the heart (upper limb and scalp PICCs) or beyond the IVC-RA junction into the heart (lower limb PICCs). Central to non-central (Outward): The tip moved outside the SVC or IVC. Outward migration was further classified as near-central (e.g., the tip positioned between SVC and the head of the humerus for upper extremity PICCs, and between lower end of IVC and the head of the femur for lower extremity PICC) or non-near-central (e.g., situated beyond the head of the humerus or femur). Non-central to Central (Inward): The tip moved into the SVC or IVC from a non-central location, such as internal jugular, brachiocephalic, or subclavian vein. Non-central to Central (Outward): The tip moved outward from within the heart but remained within the SVC or IVC. We also considered movement from the SVC/right brachiocephalic vein to the left brachiocephalic vein, or vice versa, migration.

Satisfactory repositioning referred to successful manual adjustments that moved the PICC tip to a central or low-risk, non-central position. Low-risk non-central positions excluded flexion creases (e.g., axilla or inguinal regions); we defined them as tips in the subclavian, jugular, axillary, iliac, or femoral veins.

Complications attributed to PICCs included pericardial, pleural, and peritoneal effusion, pericardial tamponade, dysrhythmia, breakage, and leakage, as reported by staff and verified by the unit’s quality leader. We classified moderate-to-severe bronchopulmonary dysplasia, retinopathy of prematurity, necrotizing enterocolitis, and intraventricular hemorrhage using CNN definitions [16].

### 2.7. Outcome Measures

The primary outcome was the frequency of migration observed between the last X-ray after PICC insertion and the 3–7 h post-insertion X-ray. Secondary outcomes included the success rates of manual repositioning to central or low-risk, non-central positions and PICC-related complications.

### 2.8. Ethics

The study received approval from the BCCW-UBC Research Ethics Board (REB) with protocol code H22-01528.

### 2.9. Statistical Methods

We present continuous variables as medians with interquartile ranges and categorical variables as frequencies and proportions. We used chi-square tests, Student’s *t*-tests, or Mann–Whitney U tests to analyze univariate risk factors for PICC migration. Multivariate logistic regression estimated adjusted odds ratios to further evaluate significant predictors (*p* < 0.05), controlling for covariates such as birth weight, Score for Neonatal Acute Physiology with Perinatal Extension-II (SNAPPE-II) scores, and postmenstrual age at insertion. Statistical significance was set at *p* < 0.05. We performed all analyses using SPSS (IBM Corp. Released 2023. IBM SPSS Statistics for Mac, Version 29.0.2.0, Armonk, NY, USA: IBM Corp).

## 3. Results

**Figure 1:**
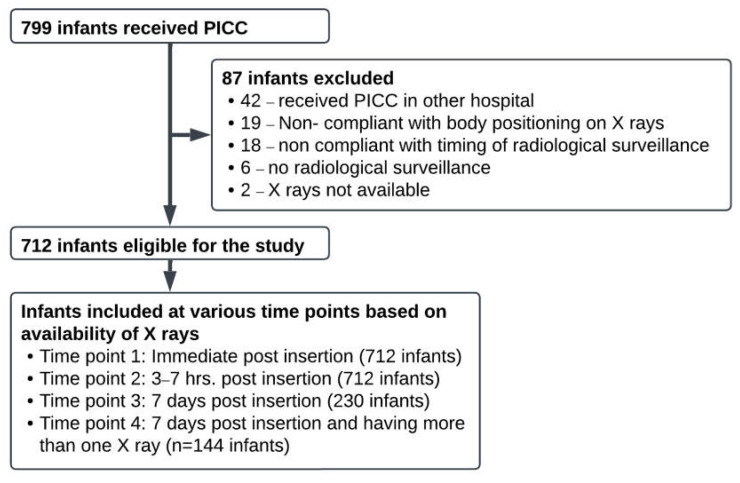
Study flow diagram.

Out of 799 infants who received a PICC, 712 met the inclusion criteria (Figure 1). The median gestational age (interquartile range) was 30 weeks (IQR 27–36), and the median birth weight was 1386 g (IQR 941–2567). The upper limb was the insertion site for most (73.5%) PICCs, and NICU staff placed 97.3% of them. We performed a median of 4 (IQR 3–5) immediate post-insertion X-rays (AP view) to assess tip location and 1 (IQR 1–1) X-ray at 3–7 h post-insertion to assess tip migration. We manually adjusted the placement in 67% of the infants immediately after insertion and in 47% after the 3–7 h post-insertion X-rays. 43.2% (268/621) of infants with upper limb or scalp PICCs received intubation and ventilation. We present additional insertion details in Table 1.

### 3.1. Frequency of Migration and Satisfactory Repositioning

Between immediate post-insertion and the 3–7 h X-ray, PICC tips migrated in 211 infants (29.6%). Most migrations (81.4%) moved inward into the cardiac silhouette (Table 2). Some non-central PICCs (n = 23) migrated into a central position because of tips originally in the cardiac silhouette returning to the central location. Of 262 non-central tips at 3–7 h post-insertion, 230 underwent manual adjustment: 216 were retracted from the cardiac silhouette to the central position, and 14 were repositioned from a non-central location to a low-risk, non-central position. The procedure resulted in a satisfactory tip position in 191 infants (83%).

Migration rates between the 3–7 h X-ray and the next X-ray taken after seven days (Time Points 2 and 3) were 22.1%, with most migrations being central to non-central. Migration between the first X-ray after seven days and the final X-ray before PICC removal (Time Points 3 and 4) was 41%, also predominantly central to non-central. In 75% of cases, we achieved satisfactory repositioning after the Time Point 3 X-ray; we performed no repositioning after the final X-ray (Time Point 4) (Table 2).

### 3.2. Factors Associated with Migration

We did not identify any significant associations between migration and infant demographics, resuscitation factors, timing of insertion, insertion side, or ventilation support at the time of insertion. However, migration occurred more frequently in infants with upper limb PICCs (82.4%), *p* = 0.0001. This association remained significant after adjusting for covariates (Table 3).

### 3.3. Adverse Events

Six PICC-related safety events required treatment during the study period: two cases of extravasation and one each of the incorrect dressing application, catheter blockage, incorrect line setup, and pleural effusion. None of these events resulted in severe complications, prolonged morbidity, or death. A quality leader’s review, along with staff consultations, determined that these events did not meet the criteria for a root cause analysis to uncover system-level weaknesses or failures.

### 3.4. Outcomes at Discharge

Of the infants who received PICCs, 94% survived, with a median length of stay of 41 days (IQR 18–85) (Supplementary Table S1). Among those requiring ventilation or supplementary oxygen, the median durations were 7 days (IQR 3–23) and 14 days (IQR 3–28), respectively. Late-onset sepsis occurred in 15% (109/712) of infants. For preterm infants born before 33 weeks, rates of moderate-to-severe bronchopulmonary dysplasia (BPD), retinopathy of prematurity (ROP) Grade 3 or higher, intraventricular hemorrhage (IVH) Grade 3 or higher, and necrotizing enterocolitis (NEC) Stage 2 or higher were 47%, 15%, 7%, and 6%, respectively.

## 4. Discussion

Despite best efforts, PICC tip migration occurred in 30% of cases within 3–7 h of placement and initiation of continuous infusion. A proactive approach, which standardized body positioning during X-rays, defined target PICC tip locations, incorporated radiological surveillance at 3–6 h, and guided manual adjustments, allowed 83% of non-central PICC tips to be repositioned to central or low-risk non-central positions. Adhering to this proactive strategy for over five years with no serious adverse events underscores its value in improving patient safety.

Within 24 h post-insertion, the frequency of PICC migration observed in our study was higher than that reported by Gupta et al. [6] (23% at 1 h, 11% at 24 h), yet lower than the rates documented by Srinivasan et al. [11] (45% at 24 h) and Acun et al. [12] (60% at 24 h, 23% between 24 h and 3 days). Inward migration in this study (81%) exceeded rates reported by others (65–68%) [11,12]. The migration rate after 7 days was higher compared to the Gupta et al. study (22% vs. 9%) [6]. In our cohort, 74% of PICCs were inserted in the upper limb, a proportion that falls within the range of 39% to 95% reported in previous studies [6,11,12]. Consistent with some reports [6,11], migration occurred more frequently in infants with upper limb PICCs, although Acun et al. [12] did not observe this association. These variations may stem from increased vigilance under the proactive approach in this study, differences in defining migration and satisfactory repositioning, and standardized body positioning for X-rays and the site of PICC insertion.

We agree with Srinivasan’s explanation for inward migration, where all migrations occurred in PICC placed in the upper limbs [11]. Securing the catheter at the insertion site on one end, continuous infusion through the PICC, and blood flow likely straighten the catheter’s tortuous course in the vein, causing the tip to move inward. However, we question the validity of migration rates between three and seven hours post-insertion and the first X-ray after 7 days, as well as between the first X-ray after 7 days and the final X-ray. We performed many of these X-rays for non-PICC-related reasons, with inconsistent infant body part positioning. X-rays were unavailable for 482 infants beyond 7 days and for 86 infants between 7 days and discharge, introducing potential selection bias. Thus, our study data cannot be used to guide the timing of serial radiological surveillance.

Previous studies did not report successful repositioning rates [6,11,12]. Unlike those studies, this study highlights the impact of intervention, achieving satisfactory repositioning in 83% of cases.

Our findings show a higher risk of migration with upper limb PICCs compared to lower limb or scalp placements, consistent with previous studies [6,11]. However, Acun et al. found no such association [12]. As with prior studies, we found no link between migration and birth weight or day of insertion [11,12].

We did not observe any instance of pericardial effusion in this study, aligning with previous reports [11,12]. Because this study focused on serious adverse events, a direct comparison of other complications was not possible.

This is the first study to investigate PICC migration in neonates at 3–7 h post-insertion, including scalp PICCs. The ideal timing of performing a surveillance X-ray is unknown [6,11,12,17]. Radiological surveillance at 3–7 h offers several advantages, such as minimizing continuous handling and allowing sufficient time for catheter stabilization. We achieved satisfactory repositioning in 83% of cases, significantly reducing risk exposure duration to a few hours instead of 24 h [3,5] for unrecognized catheter migration into the heart and potential complications. However, we must weigh these benefits against the increased X-ray exposure and handling needed to optimize limb positioning. The National Association of Neonatal Nurses (NANN) neonatal PICC practice guidelines prioritize the lower extremities as the preferred site for PICC placement. In contrast, our study predominantly used the upper limbs for PICC insertion, which may have contributed to an increased rate of PICC tip migration. The proactive approach involving multiple X-rays could cause inadvertent radiation exposure. We plan to update our unit’s PICC guidelines to incorporate NANN’s recommendations by adopting lower limb insertion and integrating point-of-care ultrasound to minimize malpositions and reduce X-ray exposure [18].

This study has several limitations. As a retrospective study, it primarily focused on PICC migration at a single time point (3–7 h post-insertion). Inconsistent definitions of “central position” and imaging protocols across the literature complicated data comparison. We relied on plain radiographs to interpret the PICC tip position, which may be less sensitive than ultrasound or fluoroscopy [13,19]. Differentiating between catheter “movement” reflecting limb positioning, potential inadequate manual adjustment immediately following placement, and true intrinsic migration remains challenging, even with adherence to positioning guidelines. A key limitation of our study is the lack of baseline data on migration rates and risk exposure duration, which restricts our ability to assess the effectiveness of the proactive approach. As a pragmatic, real-world, retrospective evaluation, standardized PICC guidelines were not implemented prior to 2016, and thus we could not collect the baseline data. 

## 5. Conclusions

Thirty percent of PICCs migrate within 3–7 h of insertion and initiation of continuous infusion, with most of them being an inward migration that might increase the risk for pericardial effusion. Manual adjustments of the PICC tip according to the “proactive approach” guideline result in 83% of non-central PICC tips being repositioned to central or low-risk non-central positions.

## Figures and Tables

**Table 1:** Details of PICC insertion.

Age at insertion, Day of life, Med (IQR)	5 (4–8)
Insertion site	
Right upper limb	213 (29.5)
Left upper limb	313 (44.0)
Right lower limb	53 (7.4)
Left lower limb	38 (5.6)
Right scalp	48 (6.9)
Left scalp	47 (6.6)
Placed by NICU staff	693 (97.3)
Placed by interventional radiology	19 (2.7)
PICC days, Med (IQR) ^#^	14 (9–27)

Values indicate n (%)unless specified. Med = Median, IQR = interquartile range ^#^ Total PICC days secondary to one or more PICCs during stay.

**Table 2:** Comparison of PICC tip position and migration rates at various time points.

TIMING of PICC Placement	Central	Non-Central
Time point 1: Post PICC-insertion and manual adjustment (n = 712)	615	97 ^1^
Time point 2: −7 h. Post PICC-insertion (n = 712) No-MIGRATION remained-Central = 427 No-MIGRATION remained-non-Central = 74 MIGRATION central to non-central = 188 MIGRATION non-central to central = 23 Total MIGRATION 211 (29.6%)	450	262 ^2^
3–7 h. Post PICC-insertion and manual adjustments Satisfactory PICC tip repositioning to central or low-risk non-central position = 191/230 (83%) ^3^	641	71
Time point 3: After 7 days Post PICC-insertion (n = 230 infant X-rays available) ^4^ No-MIGRATION remained-Central = 163 No-MIGRATION remained-non-Central = 16 MIGRATION central to non-central = 28 MIGRATION non-central to central = 23 Total MIGRATION 51 (22.1%)	186	44
After 7 days of post-PICC insertion and manual adjustments Satisfactory PICC tip repositioning to central or low-risk midline ^2^ position = 3/4 (75%) ^5^	189	41
Time point 4: Final Pre-PICC removal (n = 144) ^6^ No-MIGRATION remained-Central = 65 No-MIGRATION remained-non-Central = 20 MIGRATION central to non-central = 51 MIGRATION non-central to central = 8 Total MIGRATION 59 (41%)	73	71

^1^ = Among 97 infants with non-central PICC, 2 (0.3%), 11 (1.5%) and 84 (11.8%) infants had non-near central, Near central and Non-central inside the right atrium beyond the SVC or IVC, respectively. ^2^ = Among 262 infants with non-central PICC, 13 (1.8%), 33 (4.6%) and 216 (30.3%) had non-near central, Near central and Non-central inside the right atrium beyond the SVC or IVC, respectively. ^3^ = Out of 262 (216 inward, 46 outward) non-central tip PICCs noted on 3–7 h, surveillance X rays only 230 had manual adjustment (216 and 14 were pulled back aiming for central or non-central low-risk non-central position). ^4^ = 230 infant X rays were available, Median (IQR) day of life =8 (9–11). ^5^ = Only 4 PICCs with inward tip positioning were manually adjusted. ^6^ = 144 infant X-rays were available, Median (IQR) day of life = 24 (17–29).

**Table 3:** Characteristics of infants who had PICC migration at 3–7 h from central to non-central position. n = 615 (central placement in X-ray immediate post insertion).

	Infants Who Had PICC Migration from Central to Non-Central, n = 188	Infants Who Did Not Have PICC Migration (Remained Central) n = 427	*p* Value
Gestational age (weeks), Med (IQR)	30 (26–35)	30 (27–36)	0.10 *
Birth weight, Med (IQR)	1285 (877–2250)	1412 (947–2612)	0.08
Male	110 (58.5)	240 (56.2)	0.59
PICC insertion day of life, Med (IQR)	5 (4–9)	5 (3–8)	0.60 **
Postmenstrual age at insertion (weeks), Med (IQR)	30 (27–36)	31 (28–37)	0.08 **
Insertion site			
Upper limb	155 (82.4)	288 (67.4)	0.0001
Lower limb	16 (8.5)	68 (15.9)	
Scalp	17 (9.0)	71 (16.6)	
Insertion side—left	100 (53.2)	253 (59.3)	0.161
Inserted by NICU team	179 (95.2)	417 (97.7)	0.106
Apgar score, Med (IQR)			
1 min	5 (3–7)	6 (3–8)	0.84 *
5 min	7 (6–9)	7 (6–9)	0.34 *
Vaginal delivery	58 (30.9)	137 (32.1)	0.76
Received intubation and ventilation	131 (69.7)	305 (71.4)	0.66
Received oxygen	127 (67.6)	290 (67.9)	0.93
SNAPPE II	21(5–36)	18(0–34)	0.06

Values in each cell indicate n (%) unless specified. * GA, APGAR—parametric distribution, Student *t* test, ** nonparametric distribution. Mann–Whitney U test. SNAPPE II = Score for Neonatal Acute Physiology with Perinatal Extension-II, IQR = interquartile range, PMA—postmenstrual age, PICC—percutaneously inserted central catheter.

## Data Availability

The data presented in this study are available upon request from the corresponding author due to our REB has approved for analysis and present data in aggregate and not for individual patients to maintain privacy.

## Supplementary Materials

The following supporting information can be downloaded at: https://www.mdpi.com/article/10.3390/jcm14061875/s1, Figure S1. Body positioning and PICC tip positioning for upper limb and Scalp X rays; Figure S2. Body positioning and PICC tip positioning for lower limb X rays; Table S1. Outcome of infants with PICC.

## Abbreviations

The following abbreviations are used in this manuscript:

PICC	Peripherally Inserted Central Catheters
PIV	Peripheral Intravenous Catheters
NICU	Neonatal Intensive Care Unit
AP	Anteroposterior
SVC	Superior Vena Cava
IVC	Inferior Vena Cava
RA	Right Atrium
CNN	Canadian Neonatal Network
PACS	Picture Archiving and Communication System
SNAPPE II	Score for Neonatal Acute Physiology with Perinatal Extension-II
BPD	Bronchopulmonary Dysplasia
ROP	Retinopathy of Prematurity
NEC	Necrotizing Enterocolitis
IVH	Intraventricular Hemorrhage
REB	Research Ethics Board
