# Supplementary material for: Reducing Peripherally Inserted Central Catheter Tip Migration in Neonates: A Proactive Approach to Detection and Repositioning
Source: J Clin Med. 2025 Mar 11;14(6):1875. doi: 10.3390/jcm14061875 (PMC11942808; doi:10.3390/jcm14061875)
Supplement: Supplementary file 1 [file jcm-14-01875-s001.zip › jcm-3497348-supplementary.pdf]

Supplementary Materials

PICC - Upper Limb
Imaging Flow Diagram

Target Zone

- ➢ In the SVC region: T3 to T7 or between 3rd and 7th rib
- ➢ In the bracheocephalic vein: Medial to the sternal end of the clavicle

First Image

This film is done with patient in the insertion position with the arm abducted and elbow extended. Image in this position until line is visualized in target zone. Use contrast for non-radiopaque lines.

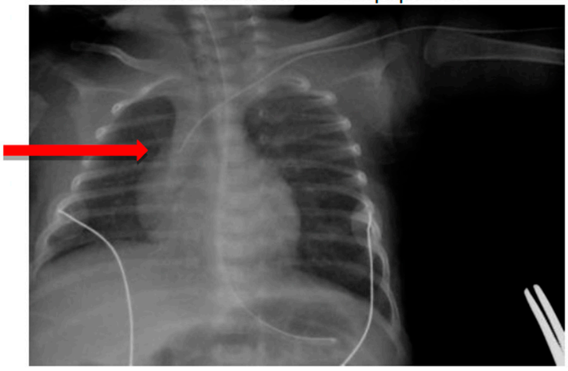

First Limb Position

Arm abducted and elbow extended

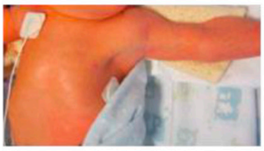

Target Zone

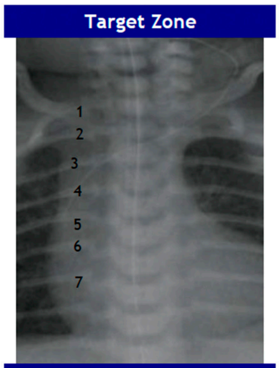

Is PICC in target zone?

No →
- Adjust line position.
- Repeat imaging in first position. until tip in target zone.
- Proceed to imaging in second position.

Yes → Second Image

Second Image

This film is done with the patient in the provocative position, with the arm adducted and elbow flexed.

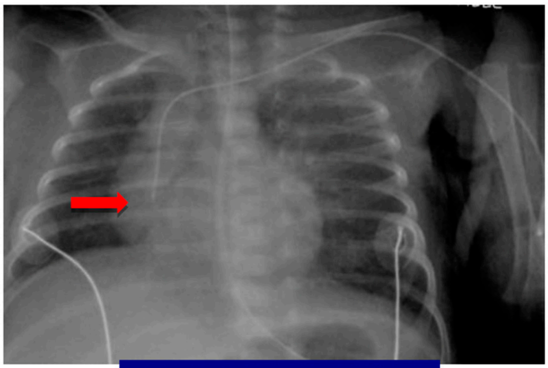

Second Limb Position

Arm adducted and elbow flexed

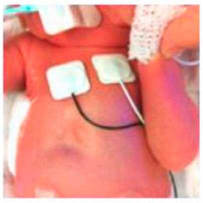

Inappropriate Limb Position

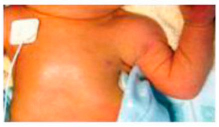

*Arm should ***not*** be lifted and rotated

Is PICC in target zone?

Yes → May use PICC

NOTE: For non-radiopaque lines, may use PICC ***except*** for vasoactive drug infusion(s).

No → Adjust PICC position. Repeat imaging in second position.

4 to 6 Hour Follow-Up Image

- Repeat image with arm positioned where the line was visualized in the deepest position. This is typically with the arm adducted (against chest) and elbow flexed.
- Use contrast for non-radiopaque lines.
- Make adjustments as required.
- May use PICC.

Supplementary Figure S1. Body positioning and PICC tip positioning for upper limb and Scalp X rays.

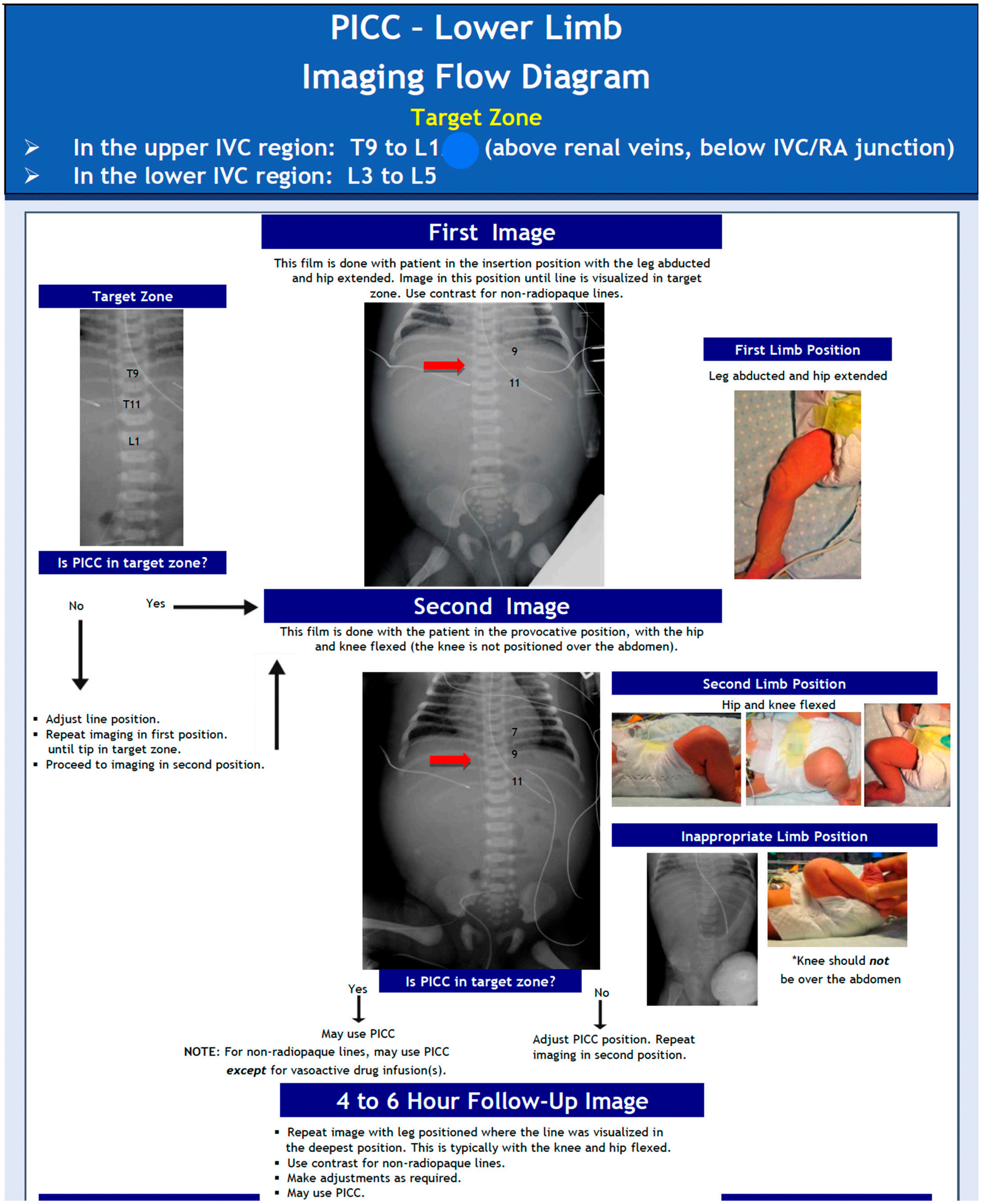

Supplementary Figure S2. Body positioning and PICC tip positioning for lower limb X rays.

Supplementary Table S1. Outcome of infants with PICC

Survival	667 (93.7)
Intubated and ventilated, Med (IQR)*	7 (3 – 23)
Oxygen days, Med (IQR) *	14 (3-48)
Late-onset sepsis	109 (15.3)
BPD **	210 (46.6)
IVH ≥ Grade 3**	34 (7.5)
NEC ≥ Grade 2**	28 (5.9)
ROP ≥ Grade 3 **	114 (15.2)
Length of stay days, Med (IQR)	41 (18.5 - 85)

Values in each cell indicate n(%) unless otherwise specified.
*Among those who received them
**<33-week infants only
